# Cannabinoid type-1 receptor signaling in central serotonergic neurons regulates anxiety-like behavior and sociability

**DOI:** 10.3389/fnbeh.2015.00235

**Published:** 2015-09-03

**Authors:** Martin Häring, Vanessa Enk, Alejandro Aparisi Rey, Sebastian Loch, Inigo Ruiz de Azua, Tillmann Weber, Dusan Bartsch, Krisztina Monory, Beat Lutz

**Affiliations:** ^1^Institute of Physiological Chemistry, University Medical Center of the Johannes Gutenberg UniversityMainz, Germany; ^2^Department of Molecular Biology, Central Institute of Mental Health, Medical Faculty Mannheim/Heidelberg UniversityMannheim, Germany; ^3^Department of Addictive Behavior and Addiction Medicine, Central Institute of Mental Health, Medical Faculty Mannheim/Heidelberg UniversityMannheim, Germany

**Keywords:** serotonin, CB_1_ receptor, raphe nuclei, sociability, anxiety

## Abstract

The endocannabinoid (eCB) system possesses neuromodulatory functions by influencing the release of various neurotransmitters, including γ-aminobutyric acid (GABA) and glutamate. A functional interaction between eCBs and the serotonergic system has already been suggested. Previously, we showed that cannabinoid type-1 (CB_1_) receptor mRNA and protein are localized in serotonergic neurons of the raphe nuclei, implying that the eCB system can modulate serotonergic functions. In order to substantiate the physiological role of the CB_1_ receptor in serotonergic neurons of the raphe nuclei, we generated serotonergic 5-hydroxytryptamine (5-HT) neuron-specific *CB*_1_ receptor-deficient mice, using the Cre/loxP system with a tamoxifen-inducible Cre recombinase under the control of the regulatory sequences of the tryptophan hydroxylase 2 gene (*TPH2-CreER*^T2^), thus, restricting the recombination to 5-HT neurons of the central nervous system (CNS). Applying several different behavioral paradigms, we revealed that mice lacking the CB_1_ receptor in serotonergic neurons are more anxious and less sociable than control littermates. Thus, we were able to show that functional CB_1_ receptor signaling in central serotonergic neurons modulates distinct behaviors in mice.

## Introduction

Serotonin (5-hydroxytryptamine, 5-HT) is present in many central and peripheral tissues, where it functions as a neurotransmitter or hormone. In the brain, 5-HT neurons are located in midbrain and brainstem areas, called raphe nuclei (Baker et al., 1991; Carkaci-Salli et al., 2011). Projections from the raphe nuclei innervate nearly every region of the central nervous system (CNS), including cortex, hippocampus, amygdala, striatum, hypothalamus, and spinal cord. This broad anatomical distribution is in line with the diverse behavioral functions which are modulated by serotonergic signaling.

A similarly wide distribution can be seen by the cannabinoid type-1 (CB_1_) receptor, a central component of the endocannabinoid (eCB) system controlling synaptic activity (Kano et al., 2009). To date, the presence of functional CB_1_ receptor has been verified in several important neurotransmitter systems, including the GABAergic, glutamatergic, noradrenergic, cholinergic and serotonergic neurons (e.g., Kathmann et al., 1999; Marsicano and Lutz, 1999; Hájos and Freund, 2002; Wallmichrath and Szabo, 2002; Monory et al., 2006; Häring et al., 2007; Ruehle et al., 2013; Ramikie et al., 2014).

Several investigations have evidenced that the eCB system can modulate the serotonergic system, suggesting a basis for new approaches for the treatment of anxiety disorders (Haj-Dahmane and Shen, 2011). Both eCBs and 5-HT have been shown to influence numerous physiological functions and to control a wide range of behaviors and emotional states (Häring et al., 2012; Ruehle et al., 2012; Asan et al., 2013; Bellocchio et al., 2013; Hanks and González-Maeso, 2013). Importantly, their functional interaction could be verified at the behavioral level. Specific cannabinoid-induced emotional changes were shown to depend on functional 5-HT transmission (Gobbi et al., 2005; Bambico et al., 2007; Häring et al., 2013).

It is believed that serotonin transmission is modulated via the activation of glutamatergic CB_1_ receptor in the dorsal raphe nucleus (DRN), with focus on the prefrontal cortex (PFC)–DRN pathway (Gobbi et al., 2005; Haj-Dahmane and Shen, 2005; Bambico et al., 2007; Haj-Dahmane and Shen, 2009). Interestingly, the systematic administration of URB597, an inhibitor of fatty acid amide hydrolase (FAAH), can enhance the firing of serotonergic neurons in DRN and increase the hippocampal levels of 5-HT (Gobbi et al., 2005). This effect is mimicked by local injection of CB_1_ receptor agonists into the PFC and blocked by PFC transection (Bambico et al., 2007). Hence, the eCB system seems to control PFC circuits which modulate the signaling toward the DRN. Furthermore, local activation of CB_1_ receptor in the DRN reduces the strength of glutamatergic inputs onto DRN 5-HT neurons, suggesting that CB_1_ receptor-positive glutamatergic synapses control serotonergic neuron activity within this region (Haj-Dahmane and Shen, 2005, 2009).

A potential direct interaction between the eCB system and the serotonergic system has already been suggested by other studies as well. Global loss of the CB_1_ receptor reduced the responsiveness of mice to the anxiolytic drug buspirone, a serotonin receptor 5-HT_1A_ agonist (Urigüen et al., 2004). *In vitro* studies showed that the release of 5-HT can be altered by the cannabinoid CB_1_/CB_2_ receptor agonist WIN552122 and by the CB_1_ receptor antagonist rimonabant in mouse cortical slices (Nakazi et al., 2000). Furthermore, we identified expression of the CB_1_ receptor in serotonergic neurons at the mRNA and protein level (Häring et al., 2007), suggesting that (endo)cannabinoids may directly regulate 5-HT transmission. Finally, the CB_1_ receptor co-localizes with the serotonin transporter (SERT) and can control frontocortical 5-HT release (Ferreira et al., 2012).

The question has remained whether the low levels of CB_1_ receptor expression we observed in serotonergic neurons are functionally important in the context of the animal’s behavior. Here, the physiological role of the CB_1_ receptor in central serotonergic neurons was analyzed by generating mutant mice with a specific loss of the *CB*_1_ receptor gene in central 5-HT neurons. To this end, floxed *CB*_1_ receptor mice (Marsicano et al., 2003) were crossed with a transgenic mouse line expressing the tamoxifen-inducible Cre recombinase under the control of the regulatory elements of the tryptophan hydroxylase-2 gene (*TPH2-CreER*^T2^; Weber et al., 2009). This mouse line allows temporal control over the recombination event, thereby avoiding potential compensatory processes caused by developmental effects. Following treatment with tamoxifen during adulthood, molecular and behavioral analyses of mutants (*TPH2-CB*_1_^−/−^) and wild-type (*TPH2-CB*_1_^+/+^) littermates were performed. Specifically, we investigated whether CB_1_ receptor-expressing 5-HT neurons in the raphe nuclei influence anxiety, social interaction, and acute stress coping.

## Materials and Methods

### Breeding Strategy

Experimental protocols were carried out in accordance with the Council Directive 2010/63EU of the European Parliament and the Council of 22 September 2010 on the protection of animals used for scientific purposes and approved by the Ethical Committee on animal care and use of Rhineland-Palatinate, Germany (reference number 23 177-07/G 08-1-021).

The conditional mutant mouse line, named *TPH2-CB_1_^−/−^* after tamoxifen-induced recombination, in which the *CB*_1_ receptor is deleted from central 5-HT neurons, was generated by a three-step process. As a first step, homozygous *CB_1_^fl/fl^* female mice (Marsicano et al., 2003) were crossed with males bearing a tamoxifen-inducible CreER^T2^ recombinase expressed under the regulatory elements of the mouse TPH2 gene (*TPH2^CreERT2^*; Weber et al., 2009). In a second step, heterozygous *CB_1_^fl/+; tph2-CreERT2tg/+^* mice were crossed with *CB_1_^fl/fl^* mice to obtain *CB_1_^fl/fl; tph2-CreERT2tg/+^* mice. At last, male *CB_1_^fl/fl; tph2-CreERT2tg/+^* mice were bred with *CB*_1_^fl/fl^ females to generate the experimental animals *CB_1_^fl/fl; tph2-CreERT2tg/+^*, after tamoxifen-induced recombination referred to as *TPH2-CB*_1_^−/−^. *CB*_1_^fl/fl^ served as littermate controls and were also treated with tamoxifen, referred to as *TPH2-CB*_1_^+/+^.

To exclude potential behavioral impacts of the *TPH2-CreER*^T2^ transgene, additional experiments were performed using heterozygous transgenic *TPH2^CreERT2tg/+^* mice and their wild-type littermates containing no transgene.

### Genotyping

Genotyping was performed by polymerase chain reaction (PCR) from tail genomic DNA using the forward primer 5′-CGGCA TGGTG CAAGT TGAAT A-3′ (G100) and the reverse primer 5′-GCGAT CGCTA TTTTC CATGA G-3′ (G101) for the detection of the *Cre* recombinase locus, yielding a 400 bp band. The floxed *CB*_1_ receptor allele was detected using a forward primer 5′-GCTGT CTCTG GTCCT CTTAA A-3′ (G50) and a reverse primer 5′-GGTGT CACCT CTGAA AACAG A-3′ (G51). Using these primers, wild-type alleles give an amplicon of 413 bp, whereas targeted (floxed) alleles give a 493 bp amplicon. The evaluation of both loci was done simultaneously in one PCR reaction (1 × 95° for 3 min; 37 × [95°C for 1 min; 54°C for 1 min; 72°C for 1 min]; 1 × 72°C for 5 min).

### Induction of Recombination

Due to the mutated ligand binding domain of the estrogen receptor (ER^T2^) fused to the Cre recombinase, tamoxifen had to be injected to allow translocation of CreER^T2^ into the nucleus, where the activated enzyme induced recombination of the floxed *CB*_1_ receptor allele. To this end, animals were intraperitoneally (i.p.) injected with either vehicle or tamoxifen (freshly prepared; 2 mg/day/mouse) once per day for 5 days at the age of 10–17 weeks. Vehicle solution consisted of 90% sun flower seed oil and 10% ethanol (Sigma Aldrich). For behavioral experiments, all animals were treated with tamoxifen to exclude any treatment induced effect.

### Evaluation of Recombination by Genomic PCR in Tissue Sections

One week after the last tamoxifen injection, mice were sacrificed by decapitation after isoflurane anesthesia. The brain and duodenum were removed. By using a brain matrix, the brain was cut into 1 mm sections. Using a 1 mm puncher, several brain regions were isolated. The duodenum was rinsed in 1 × PBS to remove digested material. For brain and duodenum tissue, DNA isolation was performed as follows: Samples were treated overnight at 56°C in 500 μl lysis buffer (100 mM Tris-HCl pH 8, 5 mM EDTA, 200 mM NaCl, 0.2% SDS) and 10 μl proteinase K (10 mg/ml stock concentration) in a shaking (800 rpm) thermomixer (Eppendorf). On the next day, the probes were centrifuged for 2 min at 13,000 rpm. The supernatant was collected and mixed by inversion with 500 μl phenol/chloroform mixture (Sigma cat P2069). Following another centrifugation step for 5 min at 13,000 rpm, the upper (aqueous) phase was transferred and mixed by inversion with 500 μl chloroform. The probes were again centrifuged for 5 min at 13,000 rpm. Subsequently, the supernatant was transferred, mixed by inversion with 500 μl isopropanol and centrifuged for 5 min at 13,000 rpm. The supernatant was completely removed, and the pellet was washed with 70% ethanol by centrifugation for 5 min at 13,000 rpm. After removing the supernatant, the pellet was air dried at RT for 10 min and dissolved in 20 μl PCR grade H_2_O. The DNA was tested for the recombination event by PCR reaction (1 × 95° for 3 min, 37 × [95°C for 1 min; 54°C for 1 min; 72°C for 1 min], 1 × 72°C for 5 min) using the forward primer 5′-GCTGT CTCTG GTCCT CTTAA A-3′ (G50) and the reverse primer 5′-CTCCT GTATG CCATAG CTCTT-3′ (G53), obtaining a 600 bp band.

### *In situ* Hybridization

In order to visualize the minute amounts of CB_1_ mRNA in central serotonergic neurons and thus verify successful recombination *in situ* in *TPH2-CB*_1_^−/−^ mice, we employed a highly sensitive *in situ* hybridization (ISH) system (ViewRNA^TM^ ISH Tissue 2-Plex Assay; Affymetrix). Four weeks after tamoxifen injections, mice were sacrificed and brains were isolated. Three mm thick tissue blocks were cut using a brain matrix approximately from −3.5 to −6.5 mm from the bregma. Tissue was fixed with 4% paraformaldehyde for 16–24 h, rinsed, dehydrated and embedded in a paraffin block. Paraffin-embedded tissue blocks were sectioned at a thickness of 6 μm and mounted on Fisherbrand Superfrost Plus Slides (Thermo Scientific), air-dried at 37°C for 5 h and stored at 4°C. Slides were baked at 60°C overnight and then dewaxed in xylene. Following heat pretreatment with pretreatment solution at 85–90°C for 10 min, the tissue was digested with Protease QF (Affymetrix) for 10 min and then hybridized with QuantiGene ViewRNA probes (Mouse Cnr1 Type 1, NM_007726, VB1-10422; Mouse Tph2 Type 6, NM_173391, VB6-16623; Mouse Ubc Type 1, NM_019639, VB1-10202; Affymetrix). Bound probes were pre-amplified and subsequently amplified according to the manufacturer’s guidelines. Probe oligonucleotides of Label Type 1 conjugated to alkaline phosphatase were added followed by Fast Red substrate. Subsequently, probe oligonucleotides of Label Type 6 conjugated to alkaline phosphatase were added followed by the Fast Blue substrate. Afterwards, slides were cover-slipped with Mowiol mounting medium. Images were acquired using a Leica DM5500B microscope (Leica).

### General Outline of Behavioral Experiments

Animals were group housed (3–5 animals per cage) in a temperature- and humidity-controlled room with a 12 h light-dark cycle (lights on at 1 am). Adult males were treated with tamoxifen (2 mg/day for 5 days i.p.) between 10–17 weeks of age to induce recombination in order to obtain *TPH2-CB*_1_^+/+^ and *TPH2-CB*_1_^−/−^ mice. An interval of at least 3 weeks ensured the degradation of the CB_1_ receptor transcript and protein and prevented stress-induced behaviors due to tamoxifen injection (Vogt et al., 2008). In the 3rd week, mice were single housed to avoid behavioral differences between dominant and subordinate animals. Behavioral analyses started 4 weeks after the treatment. If not stated otherwise, experiments were performed 1 h after turning off the lights (at 2 pm) in the active phase of the animals, with only a dim red light source in the room (0 lux).

After each test, the equipment used was cleaned with 70% ethanol and then with water. Experiments were recorded on DVD, and the behavioral performance was evaluated by hand (EPM, FST, LD, NORT, NSF and RI) or SMART software (Panlab, Spain; Sociability and Open field, OF).

### Behavioral Tests

Animals were tested in a battery of behavioral tests, which were performed in the order in which they are described below. The tests Open field, Light Dark (LD), Novelty Suppressed Feeding (NSF), Marble Burying and Elevated Plus Maze (EPM) were performed within 1 week, always with 24 h in between the tests. In order to minimize the potential effect of stress due to the test battery, animals were left undisturbed for 1 week before continuing with the RotaRod test and the Forced Swim Test (FST). For the Social and Object interaction paradigms, separate batches were used.

#### Open Field

To evaluate locomotor activity, an open field test was performed. The test was conducted in a plastic open field chamber (41 cm × 42 cm × 40 cm [Height × Width × Length]) and total distance moved, distance in periphery vs. center and time in periphery vs. center were measured.

#### Light Dark Test

By running the light dark test (LDT), basic anxiety-like behavior was investigated using a box with one open white compartment (40 × 26 × 38.5 cm [H × L × W]) and one closed black compartment (40 × 13 × 38.5 cm [H × L × W]) connected via an entrance. The animal was allowed to move freely between the two compartments. Experiments were performed during the light phase (200 lux above floor of lit compartment). The animal was placed directly in front of the entrance in the lit compartment. As soon as the animal had entered the closed compartment, a 5 min test period was started. The entries into the lit compartment, the time spent in the lit compartment, as well as risk assessment behavior, were evaluated. Risk assessment includes a stretch-attend posture in which the head and fore-paws of the animal extend into the lit area but the remainder of the body stays in the dark compartment (Bailey and Crawley, 2009).

#### Novelty Suppressed Feeding

The NSF paradigm measures basic anxiety-like behavior with acute food intake and body weight development (Bodnoff et al., 1989). The test was performed in a brightly lit (450 lux) white plastic box (40 cm × 80 cm × 80 cm [H × W × L]). The floor was covered with a thin layer of saw dust. To avoid disturbing the light-dark cycle, this test was performed during the light phase. A food pellet was placed on an 18-cm filter paper in the center of the box. When the test started, the animal had been food deprived for 24 h, in order to increase the drive towards the food pellet presented. The animal was placed into the box for a maximum time of 10 min. Time was measured until the animal first touched the food pellet and until the mouse started eating. When the animal had started eating, it was placed back into the home cage with food ad libitum. Food consumption was measured at different time points (5 min, 30 min, 1 h, 2 h, 4 h, 8 h and 24 h after returning to home cage).

#### Marble Burying Test

This test is assessing the anxiety response to novel stimuli (here: marbles) without the possibility of retreat. A standard cage was filled with 3–4 cm of saw dust. The test mouse was placed into the cage where 12 marbles had been evenly distributed on the saw dust (Broekkamp et al., 1986). The animal was allowed to move freely in the cage for 15 min. At the end of the test, the number of buried marbles was recorded. A high number of marbles buried is regarded as increased anxiety behavior.

#### Elevated Plus Maze

This assay is measuring anxiety in mice. The EPM, a crossed platform of two closed and two open arms made of white plastic, is 100 cm above the ground. All four arms are of 35 cm length and 6 cm width. The closed arms have additional black plastic walls of 20 cm height. The animal was placed into the center of the EPM and was allowed to explore the maze freely for a 5 min period. Time spent on the different arms and arm entries were measured.

#### RotaRod

To evaluate motor skills, animals were tested on the RotaRod apparatus from Ugo Basile (38 × 32 × 44 cm [H × L × W]) during a 5 day protocol. The speed of the rod accelerated from 4–50 rpm (max. speed was reached after 180 s. Time and speed until the animal fell down were registered setting a 210 s cutoff time. Mice were tested three times a day with resting intervals of 5 min between the trials.

#### Sociability Test

This test was performed using a modified protocol taken from Moy et al. (2004). In short, the test chamber (41 cm × 42 cm × 70 cm [H × W × L]) was divided into three compartments (40 cm × 40 cm × 22 cm [H × L × W]), all accessible by openings (7.5 cm × 10 cm [H × W]) in the dividing walls. Chambers and cages were cleaned with 70% ethanol between each trial to avoid olfactory cues. The total time the test animals spent in each of the compartments during the sociability and social novelty phase was measured. Male C57BL/6N animals (10–12 weeks old) were used as interaction partners in both of the test phase. *Habituation Phase*: The test animal was placed into the middle compartment for 5 min with entries to the side compartments blocked. *Sociability Phase*: After the habituation phase, the blockades of the entries were removed, allowing free access to the side compartments. The test animal was thereby exposed to a novel C57BL/6N interaction partner and a novel object (round cage described below) each positioned in one of the two side compartments. The position of the interaction partner (left vs. right compartment) was alternated between trials to avoid any bias. The interaction partner itself was enclosed in a round cage (10 cm in diameter; 30 cm high [upper 20 cm Plexiglas, lower 10 cm covered by metal bars 1 cm apart to allow interaction but prevent fighting]). To minimize stress levels of these animals, they were habituated to the cages four times for 10 min. Furthermore, they were equally used for wild-type and mutant test mice to counterbalance individual behavioral differences.

#### Novel Object Recognition Task

The novel object recognition task (NORT) combines a general exploration test with a visual recognition memory paradigm. The test was performed in a white plastic open field chamber (40 cm × 40 cm × 40 cm [H × W × L]). The protocol used was modified after Ennaceur and Delacour (1988), Tang et al. (1999) and Tordera et al. (2007). For habituation, the animals were placed into the empty open field and allowed to explore the box for 10 min once a day for 2 days. On day three, two identical objects (O1 left, and O1 right; two metal cubes with 4 cm × 3 cm × 5 cm dimensions [H × W × L]) were placed symmetrically 6–7 cm from the walls with a separation distance of 16–18 cm from each other. The mouse was placed into the box at an equal distance from both objects and it was video-recorded for 10 min. After this first exposure to the object, the mouse was returned to its home cage. 2 h and 24 h later, the mouse was placed into the open field again. During these 10 min retention trials, it was exposed to the familiar object (O1), as well as to a novel object (O2 for the 2 h time point, and O3 for the 24 h time point, respectively). The novel object O2 was a plastic billiard ball (5.72 cm in diameter) fixed on a metal plate (0.2 cm [H]), O3 was a round glass flask (6 cm × 3 cm [H × W]) filled with sand and sealed with a black rubber plug. The familiar object was always positioned on the left side, while the new object was on the right side. Box and objects were cleaned with 70% ethanol after each trial to avoid olfactory cues. The experiment was video-recorded and the total time that the animal spent exploring each of the two objects in training and retention phase was evaluated by an experimenter blind to the genotype. Object exploration was defined by orienting the nose directly to the object at a distance <2 cm and/or touching the object with nose and whiskers. Time spent climbing and sitting on the object was not regarded as exploration as it rather represents a form of environment investigation. Therefore, it was excluded from measurement (Ennaceur and Delacour, 1988).

#### Resident Intruder Test

This paradigm allows evaluation of social exploration and aggressive behavior (Goyens and Noirot, 1975). The resident-intruder test was performed by placing a novel, group housed intruder into the home cage of the test animal for 10 min. To decrease interaction induced by the intruder, younger animals (males, 11–13 weeks of age) were used as intruders. The experiment was video recorded, and the total interaction time of the animals spent exploring was measured by an experimenter blind to the genotype. Interaction was defined by any type of physical interaction clearly directed towards the partner. Duration and number of fights were evaluated separately. Fighting was defined by physical struggling between the interaction partners, initiated by an attack of the resident towards the intruder.

#### Forced Swim Test

The paradigm was performed in a round glass beaker (18 cm in diameter; 35 cm [H]) filled with tap water at 25°C +/−0.5°C. The water level was approximately 20 cm to prevent the animal from touching the bottom of the glass or climbing off of the beaker. The animal was carefully lowered into the water and recorded on DVD for 6 min. The first 2 min were not evaluated; however, floating behavior was scored for the following 4 min by an experimenter blind to genotype. Floating was defined by immobility of the animal with only minimal movements to keep body balance.

### Corticosterone Measurements

5-HT is known to influence the hypothalamus-pituitary-adrenal axis. Thus, plasma corticosterone levels were measured in non-stressed (immediately killed after removal from home cage) and stressed (killed 5 min after FST paradigm) animals (Steiner et al., 2008). Mice were decapitated between 3 pm and 5 pm, and the blood was collected in EDTA di-calcium containing collection tubes (KABE Labortechnik, Nümbrecht). Plasma was separated from blood cells by centrifugation at 13,000 rpm for 5 min at 4°C and was stored until use at −20°C. Corticosterone concentration was analyzed using the Corticosterone EIA Kit (IBL, Hamburg) and measured by the FLUOstar Galaxy from BMG Labtech.

### Statistical Analysis

Data are presented as mean ± standard error of the mean (SEM) of individual data points. Results were considered to be significant at *p* < 0.05. Behavioral endpoints were initially analyzed using unpaired Student’s *t*-test or two-way ANOVA, using genotype and object as factors and Bonferroni post-tests to correct for multiple comparisons.

## Results

### CB_1_ Receptor Gene Inactivation in Serotonergic Neurons of the Raphe Nuclei

In order to induce a deletion of the *CB*_1_ receptor gene in central serotonergic neurons, *CB_1_^fl/fl^* mice were crossed with the *TPH2^CreERT2tg/+^* transgenic mice. Experimental animals, lacking CB_1_ in 5-HT neurons after tamoxifen treatment, were named *TPH2-CB*_1_^−/−^ and their control *CB*_1_^fl/fl^ littermates were named *TPH2-CB*_1_^+/+^.

**Figure 1:**
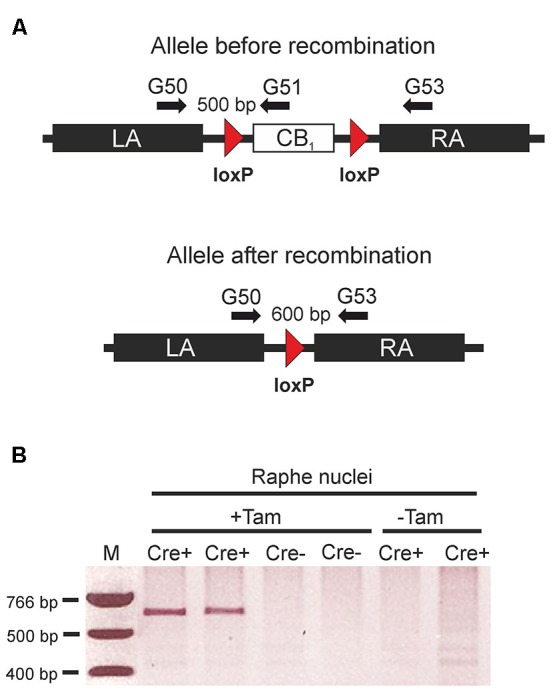
**Tamoxifen-inducible genetic inactivation of cannabinoid type 1 (CB_1_) receptor in TPH2-positive neurons. (A)** Schematic illustrations of CB_1_*^fl/fl^* allele before (above) and after Cre recombinase-mediated recombination (below). Arrows indicate primer positions. **(B)** Applying primers G50/G53, polymerase chain reaction (PCR) reactions were run using genomic DNA from the raphe nuclei region as a template. The 600 bp band indicating successful recombination was only detected in mice containing the CreER^T2^ transgene. (Cre+) after treatment with tamoxifen (+Tam). LA, left homology arm; RA, right homology arm; Cre+, animals heterozygous for the CreER^T2^ transgene and homozygous for the CB_1_*^fl/fl^* allele. Cre- = CB_1_*^fl/fl^* mice without the CreER^T2^ transgene; +Tam, tamoxifen-treated; −Tam, vehicle-treated; M, DNA size marker.

Deletion of the *CB*_1_ receptor gene in *TPH2-CB*_1_^−/−^ mice was detected only in genomic DNA isolated from the area of the raphe nuclei, but not from other brain regions or duodenum (Bellocchio et al., 2013), indicating a region-specific loss of the *CB*_1_ receptor gene. Therefore, we have isolated genomic DNA from brain punches of the raphe nuclei region of tamoxifen-treated and vehicle-treated *TPH2-CB*_1_^−/−^ and *TPH2-CB*_1_^+/+^ mice, respectively. Using primers located on the left and right homology arms (G50 and G53; Figure 1A), incidence of successful recombination events was tested by the presence of a 600 bp band. We were only able to detect this band in mice that expressed Cre recombinase in 5-HT neurons (*TPH2-CB*_1_^−/−^) and were treated with tamoxifen (Figure 1B), indicating the absence of background recombination without tamoxifen treatment in these mice.

**Figure 2:**
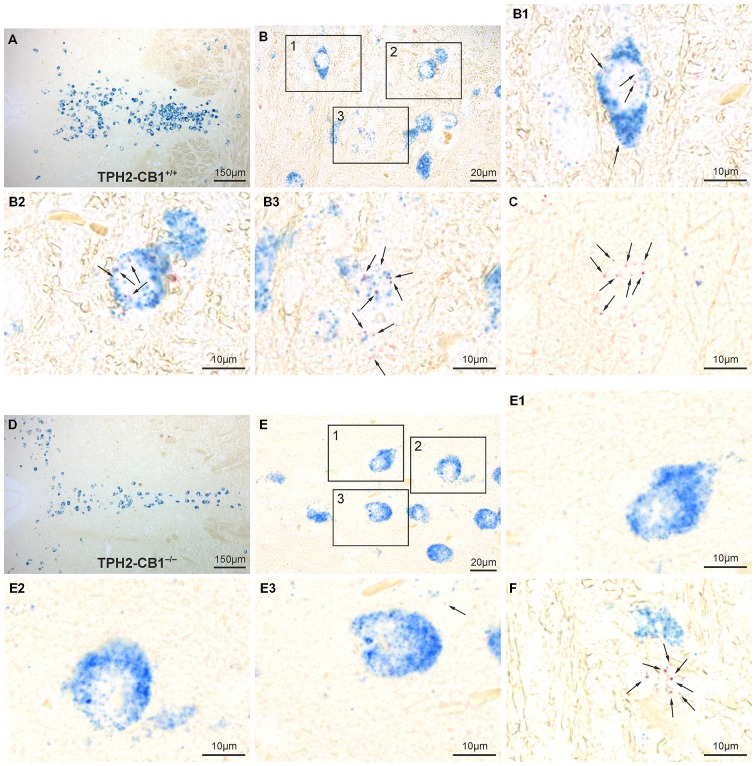
**Verification of 5-hydroxytryptamine (5-HT) neuron-specific CB_1_ loss by *in situ* hybridization (ISH). (A,D)** Overview of the dorsal raphe nucleus (DRN) double-stained for CB_1_ (red staining) and TPH2 (blue staining) in *TPH2-CB_1_^+/+^* **(A)** and *TPH2-CB*_1_^−/−^ **(D)** mice. *TPH2* probes were used to define serotonergic neurons. **(B,E)** Higher magnification of coronal hindbrain sections showing presence or absence of *CB*_1_ mRNA in serotonergic neurons in *TPH2-CB*_1_^+/+^ **(B)** and *TPH2-CB*_1_^−/−^ **(E)** mice. Note that the low expression levels of *CB*_1_ (2–7 red dots in a cross-section of a serotonergic neuron) make visualization of coexpression possible only at high magnification (see inserts). In *TPH2-CB*_1_^−/−^ mice, less than 1 in 100 serotonergic cell expressed *CB*_1_. **(B_1_–B_3_)** Serotonergic neurons expressing *CB*_1_ mRNA and **(C)** a *CB*_1_-positive, non-serotonergic neuron in *TPH2-CB*_1_^+/+^. **(E_1_–E_3_)** Serotonergic neurons are devoid of *CB*_1_ mRNA in *TPH2-CB*_1_^−/−^ mice, but *CB*_1_-positive, *TPH2*-negative neurons **(F)** are still present. Arrows, Fast Red alkaline phosphatase substrate, representing *CB*_1_ mRNA localization.

Cell-type specific deletion of the *CB*_1_ receptor gene in *TPH2-CB*_1_^−/−^ mice was further verified by ISH with *CB*_1_ and *TPH2* probes (Figure 2). *TPH2* probes were used to define serotonergic neurons. In accordance with our previous study (Häring et al., 2007), we found *CB*_1_ receptor mRNA in a subpopulation of serotonergic neurons in *TPH2-CB*_1_^+/+^ mice. Furthermore, the expression levels for *CB*_1_ mRNA were very low in this cell population. Tamoxifen-induced recombination resulted in a ≥90% decrease of the number of *CB*_1_-positive serotonergic neurons in *TPH2-CB*_1_^−/−^ mice.

### No Alterations in Locomotor Activity and Motor Skills

The evaluation of motor skills in the RotaRod test revealed that both genotypes improved their performance during the 5 days of training, but there were no differences between genotypes (Time spent on rod on day 5: 101.07 ± 6.169 s for *TPH2-CB_1_^−/−^* [*n* = 23] and 85.57 ± 7.69 s for *TPH2-CB*_1_^+/+^ [*n* = 24]; genotype effect [*F*_(1,630)_ = 0.61; *p* = 0.4384]; time effect [*F*_(14,630)_ = 40.02; *p* < 0.001]). Moreover, no difference was observed for the locomotor activity in the open field and in the sociability test (Open Field: 2899 ± 92.35 cm for *TPH2-CB*_1_^−/−^ [*n* = 42] and 2846 ± 118.4 cm for *TPH2-CB*_1_^+/+^ [*n* = 50]; [*T*_(90)_ = 0.3604; *p* = 0.719]; Sociability-Habituation: 930.7 ± 48.96 cm for *TPH2-CB*_1_^−/−^ [*n* = 18] and 1096 ± 53.47 cm for *TPH2-CB*_1_^+/+^ [*n* = 201]; [*T*_(369)_ = 1.91337; *p* = 0.0637], Sociability phase: 4436 ± 82 cm for *TPH2-CB*_1_^−/−^ [*n* = 18] and 4680 ± 167 cm for *TPH2-CB*_1_^+/+^ [*n* = 21]; [*T*_(37)_ = 0.1458; *p* = 0.1539]).

### No Alterations in Basal Anxiety-Like Behavior

**Figure 3:**
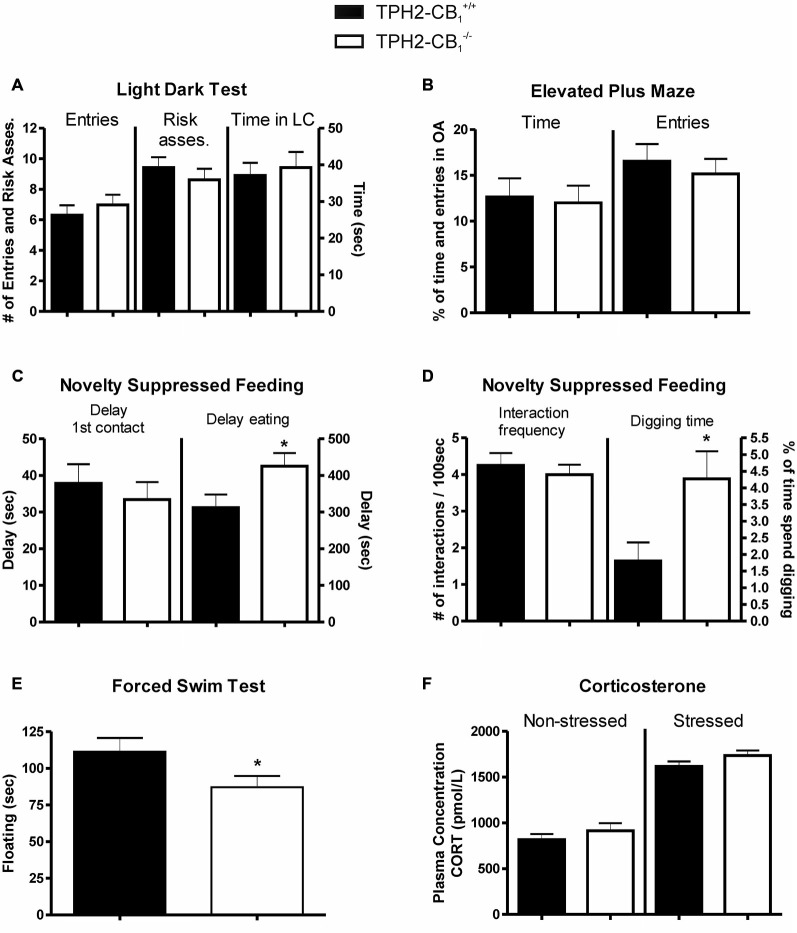
**Anxiety and stress response.** No genotype differences were observed in basic anxiety tests, such as **(A)** the light dark test (LDT) and **(B)** the elevated plus maze (EPM). **(C)** Increased aversive environment in the novelty suppressed feeding (NSF) test induced anxiety-like behavior in *TPH2-CB1^−/−^* mice. Although no genotype differences were observed in the time required for the first contact, mutants showed a delayed time to start eating. **(D)** While the frequency of interactions with the food pellet was not altered, a significant increase in the percentage of time spent digging was observed. **(E)** Mutants displayed a decreased floating behavior in the forced swim test (FST) as compared with control littermates. **(F)** No genotype differences were observed in plasma corticosterone concentrations, neither in basal conditions (non-stressed) nor 5 min after the FST (stressed). Risk asses = risk assessment; LC = lit compartment; *n*_[*EPM+LD*]_ = 41 WT + 49 KO; *n*_[*NSF*]_ = 32 WT + 33 KO; *n*_[*FST*]_ = 20 WT + 22 KO; *p < 0.05.

The basic anxiety response was evaluated using the LDT and EPM, two established and acknowledged anxiety paradigms. However, none of these tests revealed any genotype differences. In the LDT, risk assessment, entries into the lit compartment, and the time spent in the lit compartment were not different in the *TPH2-CB_1_^−/−^* as compared to *TPH2-CB*_1_^+/+^ littermates (entries [*T*_(88)_ = 0.2126; *p* = 0.8321]; risk assessment [*T*_(88)_ = 0.1184; *p* = 0.9060]; time spent in the lit compartment [*T*_(1,88)_ = 0.5223; *p* = 0.6028]; Figure 3A). Similarly, no differences between genotypes were detected in the EPM regarding the percentage of time spent and entries on the open arm (time [*T*_(88)_ = 0.2288; *p* = 0.8195]; entries [*T*_(88)_ = 0.5616; *p* = 0.5758]; Figure 3B). Furthermore, in the marble burying test, likewise an anxiety paradigm, no difference in the number of marbles buried was observed (4.531 ± 0.6350 marbles for *TPH2-CB*_1_^−/−^ [*n* = 32] and 5.938 ± 0.7239 marbles for *TPH2-CB*_1_^+/+^ [*n* = 32]; *T*_62)_ = 1.490; *p* = 0.1492).

### Increased Aversive and Social Stimuli Lead to a Reduced Animate Exploration, Anxiety-Like Behavior, and Aggravated Stress Coping

In contrast to the basal anxiety as evaluated in the LDT and EPM, increasing the aversiveness of the environment by bright illumination or inescapable stress as well as applying a social exploratory stimulus led to behavioral alterations in the *TPH2-CB_1_^−/−^* mice as compared to the *TPH2-CB*_1_^+/+^ littermates.

Even though the first contact with the food pellet was not altered in the NSF paradigm, the *TPH2-CB_1_^−/−^* mutants showed an increased latency to start eating the food pellet (delay of first contact [*T*_(44)_ = 0.5774; *p* = 0.5666]; delay of eating [*T*_(44)_ = 2.112; *p* < 0.05]; Figure 3C). Moreover, the *TPH2-CB*_1_^−/−^ animals displayed an increase in digging events and time spent digging, but no alterations in interaction frequency (digging events [*T*_(43)_ = 2.774; *p* < 0.01]; time spent digging [*T*_(43)_ = 2.465; *p* < 0.05]; Figure 3D). At the same time, food consumption in the first 5 min directly after the experiment did not differ between wild types and mutants (*TPH2-CB*_1_^+/+^: 0.1762 ± 0.02264 g, *n* = 21; *TPH2-CB*_1_^−/−^: 0.2070 ± 0.02789 g, *n* = 23; *p* = 0.4020).

The inescapable stress in the FST induced a significant decrease in time spent immobile, termed as floating behavior (*T*_(42)_ = 2.033; *p* < 0.05; Figure 3E). To address whether the hypothalamus-pituitary-adrenal axis is affected, we also measured corticosterone levels in unstressed (no FST) and stressed conditions (5 min after FST). Stress increased the corticosterone levels significantly in both genotypes (*TPH2-CB1*^−/−^ [*T*_(17)_ = 9.892; *p* < 0.001]; *TPH2-CB1^+/+^* [*T*_(18)_ = 8.490; *p* = 0.001]; Figure 3F). However, no significant differences between genotypes were observed in either of the two conditions (unstressed [*T*_(17)_ = 0.9445; *p* = 0.3581]; stressed [*T*_(18)_ = 1.511; *p* = 0.1482]; Figure 3F).

**Figure 4:**
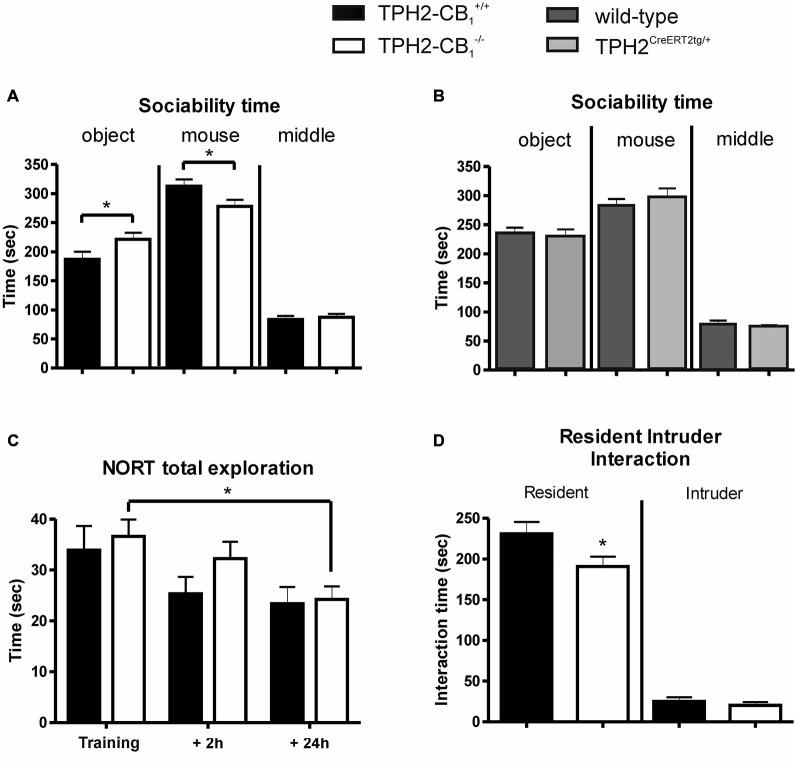
**Social and object exploration. (A)** In the sociability test, social exploration was reduced in mutant animals, whereas object exploration was increased as compared with control littermates. **(B)** In contrast to *TPH2-CB_1_^−/−^* mice, mice only possessing the CreER^T2^ transgene (TPH2*^CreERT2tg/+^*) did not show any behavioral alterations in the sociability paradigm as compared to their wild-type littermates. **(C)** No genotype differences were observed in the novel object recognition test (NORT), as both groups spent an equal amount of time exploring the objects. **(D)** The resident intruder (RI) test revealed that the social interaction initiated by mutant mice was reduced compared to control littermates. This reduction did not depend on the behavior of the C57BL/6N intruder as intruders showed the same interaction time towards both groups of residents. *n*_[Sociability]_ = 18 WT+ 21 KO; *n*_[NORT]_ = 12 WT + 11 KO, *n*_[RI]_ = 30 WT + 30 KO; *n*_[CreERT2]_ = 14 WT + 15 KO; 2-Way ANOVA with Bonferroni’s *post hoc* test; *p < 0.05.

The introduction of a social interaction partner in the sociability paradigm revealed a decrease in the time spent in the compartment with the interaction partner for the *TPH2-CB_1_^−/−^* mice as compared with their *TPH2-CB*_1_^+/+^ control littermates (*T*_(37)_ = 2.339, *p* < 0.05; Figure 4A). While the time spent in the middle compartment did not differ between genotypes, mutant mice spent significantly more time in the compartment with the empty cage, i.e., with the object (object [*T*_(37)_ = 2.220, *p* < 0.05]; middle [*T*_(37)_ = 0.4305, *p* = 0.6693]; Figure 4A). This is underlined by the significant difference between the two genotypes regarding social preference (*T*_(37)_ = 2.367, *p* < 0.05).

Next, we performed the same test with heterozygous *TPH2^CreERT2tg/+^* and their wild-type littermates in order to test a potential behavioral effect induced by the CreER^T2^ transgene. No difference was observed between the genotypes for the time spent in the different compartments (Figure 4B), excluding the possibility that the behavioral alterations observed above in *TPH2-CB*_1_^−/−^ mice are caused by the Cre transgene.

The analyses of the NORT revealed no genotype differences, neither in general object exploration nor in object recognition (object exploration [*F*_(1,63)_ = 0.6438; *p* = 0.4253]; object recognition [*F*_(1,63)_ = 1.459; *p* = 0.2316]; Figure 4C). Time of exploration significantly decreased over the three phases of the NORT (*F*_(2,63)_ = 5.243; *p* < 0.01). However, Bonferroni post-test analysis revealed only a significant difference for the *TPH2-CB1*^−/−^ mice when comparing training and 24 h retention phase (*T*_(63)_ = 2.413; *p* < 0.05). A similar situation was observed for object recognition as time significantly affected the recognition index (*F*_(2,63)_ = 15.81; *p* < 0.001). Here, Bonferroni post-test analysis showed significant changes for both genotypes, *TPH2-CB*_1_^−/−^ (training vs. 24 h [*T*_(63)_ = 3.432; *p* < 0.01) and *TPH2-CB*_1_^+/+^ (training vs. 24 h [*T*_(63)_ = 4.545; *p* < 0.001]; 2 h vs. 24 h [*T*_(63)_ = 2.494; *p* < 0.05]).

Similarly to the sociability test, in the resident intruder (RI) paradigm, *TPH2-CB_1_^−/−^* mice (residents) also displayed a significantly decreased time interacting with the social interaction partner as compared with their control wild-type littermates (interaction-absolute time [*T*_(56)_ = 2.178; *p* < 0.05]; Figure 4D). No difference was observed in the interaction induced by the intruder (interaction-absolute time [*T*_(56)_ = 0.9704; *p* = 0.3360]; Figure 4C). There were no genotype differences detectable regarding the number of fights (0.933 ± 0.3864 for *TPH2-CB*_1_^−/−^ [*n* = 30] and 1.286 ± 0.4766 for *TPH2-CB*_1_^+/+^ [*n* = 28]; *T*_(56)_ = 0.5779; *p* = 0.5657) and the delay until the first fight occurred (539.4 ± 22.55 s for *TPH2-CB*_1_^−/−^ [*n* = 30] and 487.9 ± 35.2 s for *TPH2-CB*_1_^+/+^ [*n* = 28]; *T*_(56)_ = 1.249; *p* = 0.2160).

## Discussion

In this study, we demonstrated that specific deletion of CB_1_ receptor in central serotonergic neurons leads to distinct behavioral alterations, which emerged only under stressful situations, while no behavioral differences were observed in basal locomotion and anxiety paradigms under low levels of stress.

More aversive environments or inescapable stress as displayed in the NSF paradigm and FST, respectively, induced subtle but significant behavioral changes. In the NSF paradigm, *TPH2-CB_1_^−/−^* mice required more time to start eating and spent more time digging as compared with control mice. The NSF paradigm was established as an anxiety test to measure the rodent’s aversion to eat in a novel environment. The test is sensitive to acute administration of anxiolytic drugs, such as benzodiazepines, which induce a decreased delay in approaching and eating the food pellet (Belzung et al., 1987). Additionally, the increased digging behavior observed for the *TPH2-CB*_1_^−/−^ mice might indicate a stereotypic response in this stressful situation to the high aversive and emotional distress (Pietropaolo et al., 2011). An alternative explanation for this phenotype could be a decreased drive to eat in *TPH2-CB1*^−/−^ mice. However, we could not observe any differences in the immediate food intake after the NSF between wild types and mutants.

In contrast, the decreased time spent immobile in the FST might be regarded to be an anti-depressive-like behavior, as antidepressant drugs induce higher mobility in this test (Borsini and Meli, 1988; Petit-Demouliere et al., 2005). However, this particular effect might be caused by multiple underlying mechanisms (Petit-Demouliere et al., 2005). One explanation might be an unbalanced stress coping based on an increased fear level. In fact, the parallel presence of increased anxiety-like and anti-depressive-like responses in the FST was previously seen in *5-HT_1A_* receptor deficient mice (Heisler et al., 1998) and in mice lacking the CB_1_ receptor in cortical glutamatergic neurons (*Glu-CB_1_*^−/−^; Steiner et al., 2008; Jacob et al., 2009; Häring et al., 2011, 2012, 2013). Therefore, the decreased immobility time in the FST may rather be interpreted as an alteration in stress coping than as an anti-depressive-like behavior. However, it is interesting to note that plasma corticosterone levels after FST were not higher in *TPH2-CB*_1_^−/−^ mice than in control mice, which constitutes a similar phenotype as reported previously for the *Glu-CB_1_*^−/−^ mice (Steiner et al., 2008). An anxiety-like phenotype is apparent by the decreased time that *TPH2-CB*_1_^−/−^ mice spent with a social interaction partner in the sociability test and RI paradigm. Notably, the investigatory drive itself seems to be unaltered, as no genotype differences were observed neither in the time spent in the middle compartment of the sociability box nor in the object exploration in the NORT. Thus, these data suggest a decreased interest in a social stimulus in the *TPH2-CB*_1_^−/−^ mice, which can be interpreted as an anxiety-like phenotype. The environmental conditions appear to be very important in the behavioral outcomes observed in *TPH2-CB*_1_^−/−^ mice. While in another study *TPH2-CB*_1_^−/−^ mice showed increased anxiety in the EPM (Dubreucq et al., 2012) when performing the assay in the light phase, we did not see genotype differences when performing the assay in the dark phase. This is consistent with our notion that the phenotype of *TPH2-CB*_1_^−/−^ mice is sensitive to environmental stress conditions.

Although so far we have not been able to provide direct evidence of an increased serotonergic drive in *TPH2-CB1^−/−^* mice, the inactivation of the *CB*_1_ receptor gene presumably increases serotonergic neurotransmission in a long-term manner. This expectation appears to contradict the phenotype observed. We found that the deletion of the *CB*_1_ receptor from central serotonergic neurons alters the behavioral performance in response to stress, causing an anxiety-like and anti-social phenotype. It is well established that 5-HT is crucially involved in a multitude of behavioral responses, most importantly in emotion and social interaction (Cools et al., 2008; Waider et al., 2011). Based on the behavioral changes observed after blocking SERT function leading to increased serotonergic drive, 5-HT has normally been associated with a decrease in fear and stress response (Williams et al., 2010; Haenisch and Bönisch, 2011). However, accumulating evidence suggests more diverse effects of chronic 5-HT excess (Haenisch and Bönisch, 2011). Genetic and pharmacological inhibition of SERT can lead to an elevated anxiety level as well as to a reduced aggression in mice (Heiming et al., 2009; Jansen et al., 2010, 2011; Homberg and Lesch, 2011). It is interesting to mention that an appropriate serotonergic tone is required for normal anxiety behavior, as both excess (see above) and lack of 5-HT (Jia et al., 2014) emerge to a comparable phenotype, i.e., increased anxiety.

At the anatomical level, it can only be hypothesized how CB_1_ receptor in 5-HT neurons mediate behavioral alterations. According to our previous findings, sparse CB_1_ receptor-positive serotonergic fibers were observed in cortical areas including hippocampus and amygdala. Both regions are essential for appropriate emotional behavioral responses, coping with anxiety and stress in particular. The PFC–DRN circuits are highly complex, with identifiable cell-type specific connectivity patterns (Pollak Dorocic et al., 2014) and activity patterns that are regulated not only by 5-HT, but glutamate and γ-aminobutyric acid (GABA) as well (Celada et al., 2001). Moreover, different classes of (topologically distinct) 5-HT receptors make it possible for DRN neurons to either stimulate or inhibit their target cells (Celada et al., 2013). In addition, the molecular diversity of 5-HT neurons adds a further level of complexity to the regulation of their activity. For example, a portion of 5-HT neurons expresses vesicular glutamate transporter VGlut3 (Amilhon et al., 2010) and a subpopulation of 5-HT neurons lack the inhibitory 5-HT_1A_ autoreceptor (Kiyasova et al., 2013). All these interconnected regulatory mechanisms enable that 5-HT release in the DRN and the projection areas can be differentially regulated (Coplan et al., 2014). How also eCB mediated transmitter release adds to the complexity of this picture we are just beginning to understand. Presence of CB_1_ in the hippocampus and amygdala at 5-HT synapses (Häring et al., 2007) means that activity-related down-regulation of 5-HT release may be possible at these sites.

In summary, we could demonstrate the physiological relevance of the CB_1_ receptor in central serotonergic neurons presumably fine tuning 5-HT transmission. Our findings are in line with recent insights on 5-HT function changing the dogma that 5-HT mediates predominantly positive emotional effects. Thus, the chronic desensitization of serotonergic signaling seems to induce negative emotional changes (Canli and Lesch, 2007; Homberg and Lesch, 2011). Psychiatric disorders such as autism spectrum disorders and schizophrenia are often associated with similar behavioral changes, such as decreased social interaction, depression and anxiety. In fact, 5-HT reuptake inhibitors are often used to treat depressive and anxiety symptoms, although the clinical effectiveness has remained elusive (Williams et al., 2010). Nevertheless, these types of drugs are highly potent in the treatment of depression disorders, suggesting that psychiatric diseases might be based on more complex physiological alterations, requiring a complex intervention approach. Our data indicate a contributing role of central serotonergic CB_1_ receptor in the effects on mood and emotional states. These effects might have influenced the anxiety/depression side effects of chronic application of the CB_1_ receptor antagonist rimonabant (Moreira et al., 2009). On a more positive note, this notion also indicates that combined pharmacotherapies targeting both the serotonergic and the eCB system might potentially be applied to treat stress-related emotional disturbances.

## Author Contributions

MH, VE, AAR, SL and IRA performed the experiments and analyzed the data. TW and DB developed analytical tools. KM and BL supervised the project, conceived and designed the experiments. MH, BL and KM wrote the paper. All authors discussed the results and implications and commented on the manuscript at all stages.

## Conflict of Interest Statement

The authors declare that the research was conducted in the absence of any commercial or financial relationships that could be construed as a potential conflict of interest.
